# An automatic improved facial expression recognition for masked faces

**DOI:** 10.1007/s00521-023-08498-w

**Published:** 2023-04-01

**Authors:** Yasmeen ELsayed, Ashraf ELSayed, Mohamed A. Abdou

**Affiliations:** 1 Faculty of Science, Alexandria University, Alexandria, Egypt; 2 Faculty of Computer Science and Engineering, Alamein International University, El Alamein, Egypt; 3 Informatics Research Institute, SRTA-City, Alexandria, Egypt

**Keywords:** Convolution neural network, Feature extraction, Local binary pattern, Facial expression recognition

## Abstract

Automatic facial expression recognition (AFER), sometimes referred to as emotional recognition, is important for socializing. Automatic methods in the past two years faced challenges due to Covid-19 and the vital wearing of a mask. Machine learning techniques tremendously increase the amount of data processed and achieved good results in such AFER to detect emotions; however, those techniques are not designed for masked faces and thus achieved poor recognition. This paper introduces a hybrid convolutional neural network aided by a local binary pattern to extract features in an accurate way, especially for masked faces. The basic seven emotions classified into anger, happiness, sadness, surprise, contempt, disgust, and fear are to be recognized. The proposed method is applied on two datasets: the first represents CK and CK +, while the second represents M-LFW-FER. Obtained results show that emotion recognition with a face mask achieved an accuracy of 70.76% on three emotions. Results are compared to existing techniques and show significant improvement.

## Introduction

Covid-19 is a contagious disease that appeared at the end of the year 2019. The virus is transmitted through sneezing or coughing droplets that other people breathe in. So, the best way to protect ourselves and slow down the transmission of the virus is to wear masks and practice social distancing. The World Health Organization assured wearing masks in public and closed places to protect one’s self from infection. Studies have demonstrated that masks are an important barrier to the transmission of respiratory viruses. It is important to wear a mask or respirator correctly, ensuring a close fit on the face without gaps along the edges or around the nose, while also ensuring comfort by covering the nose and mouth. Wearing a high-quality mask or respirator may be critical in high-risk settings, for persons who are at a higher risk of developing a serious illness. Therefore, you should never go without a mask, so keep it handy when you need it. Due to the widespread use of masks, communication and emotional recognition have become more challenging as facial expressions are partially obscured.

Facial expression recognition (FER) is a crucial topic in computer vision, and it is essential for the classification of expression on face images [1]. Classification expression is used to classify various categories such as anger, happiness, fear, surprise, contempt, disgust, and sadness. Facial expressions can recognize the emotional state of people over the provided information on faces. Recognizing facial expressions while a face mask is being worn is a challenging task in the field of computer vision research [1]. This paper aims to reach a better facial expression recognition approach in masked and unmasked faces. Previous studies have shown that the use of face masks reduces the accuracy of emotion recognition and increases the time required for processing. Facial expressions are closely related to empathy and play a crucial role in facilitating social communication [2, 3].

Emotion recognition is most accurate when participants are shown full-face displays, and it was found to be less accurate if participants only see the upper region of the face [4, 5]. Since partial occlusion of the face can negatively impact emotion recognition, face masks may also affect the ability of facial expressions to accurately convey a person's emotional state [6].

Facial emotion recognition (FER) could be used in conjunction with other systems to provide multiple services, such as security issues for people making transactions through ATMs. If the camera detected fear in someone’s facial expression, it will hold this transaction [7].

A convolution neural network (CNN) has one or more convolutional layers, pooling layers, and fully connected layers and is used in image recognition [8]. The CNN is able to detect features without any human supervision. For example, some images of dogs and cats could be classified using CNNs [9]. CNN can be applied to 2D and 3D arrays of data, and it used image processing after collecting data that have different formats, i.e., natural, fake, grayscale, etc. The techniques of image processing like grayscale conversion, normalization, and data augmentation are reviewed. Grayscale images convert images to black and white images which decreased the computation complexity in machine learning algorithms [9, 10]. Normalization is referred to as data rescaling. Data augmentation is used to extend datasets, and this process includes horizontal and vertical flipping, rotation, cropping, shearing, etc. Data augmentation is necessary to obtain large datasets for CNN training [10].

The input image is used by a feature extraction network, and feature signals are used by a neural network that is extracted by classification [11]. Feature extraction is valuable for reducing redundant data from the dataset depending on the new features extracted from the image [12]. It is used to build models, increase the speed of learning, and increase the accuracy of the test dataset. There are different types of feature extraction such as local binary pattern (LBP). The LBP is an efficient method used for texture feature extraction, and this method is popular for face detection and pattern recognition approaches [13]. LBP builds binary code at every neighborhood pixel depending on the value of the center pixel. The feature selection algorithm used derives from LBP for all available images, LBP extracts features, and CNN classifies images into groups based on facial expressions. The information is converted into a binary matrix where a white pixel shows high variance, and a black pixel shows low variance [14, 15].

This paper presents a CNN aided by LBP for the extraction and classification of facial expressions on two datasets: the first compares images without masks, while the second focuses on masked images. The paper is structured as follows: the introduction is in Sect. 1, related work is presented in Sect. 2, the methodology is described in Sect. 3, the experimental results and discussion are explained in Sect. 4, and the conclusion is in Sect. 5.

## Related work

In the following paragraph, the study is about facial expression recognition without a mask: These algorithms can largely be divided into four stages: face location determination, facial landmark detection, feature extraction, and emotion recognition [16]. These stages extract key feature points, such as the mouth, nose, eyes, and eyebrows, align them, and crop the face region to a rectangle. In [17] and [18] some papers studied facial expression recognition using CNN and LBP. In Table 1 the result of previous papers is shown.

**Table 1:** Result of previous papers [17, 18]

Year	Dataset	Test accuracy
2018 [17]	CK +	90%
2021 [18]	CK +	86.7%

It uses several strategies to reach this outcome, including extracting features using LBP and subsequently CNN on a CK + dataset with seven emotions [17] where 80.30% of accuracy had been achieved for CNN and 90% accuracy for LBP feature extraction.

In [17], a study was conducted in 2018 on the CK+ dataset (which contains seven emotions) to train a CNN using LBP-based feature extraction. The test accuracy was 90% when LBP was used and 80.30% when CNN was used without LBP. [18], conducted in 2021, used preprocessing (face detection and recognition) on the CK+ dataset with Dlib, followed by LBP to capture fine details, and finally trained an SVM classifier on the extracted features. The test accuracy was 86.7%, which is lower compared to the 90% accuracy achieved in the model that combined CNN and LBP.

Zhang et al. proposed an end-to-end deep learning model that allows the synthesis of simultaneous facial images and enhances facial expression recognition by exploiting the geometric shape of the face image [19].

The algorithm of this paper can be divided into three directions: the geometric feature extraction method, the appearance feature extraction method, and the deep learning-based method [18, 20]. Input images are first preprocessed for face detection and extraction using the Dlib library. LBP feature extraction is then performed to classify emotions from the CK + dataset, which has 7 emotions [18]. Recently, deep learning is used in computer vision tasks such as facial expression recognition and achieved progress. The facial expression features are learned by convolutional neural networks (CNN). Convolution neural networks can overcome these challenges, but LBP is used to get better results than CNN [21, 22].

FER typically deals with non-occluded faces [18], but due to the spread of Covid-19, efforts have been made to address the problem of occluded faces. The FER dataset is not designed for facemask-aware recognition, so the proposed solution is the automatic wearing face mask (AWFM) method [23, 24]. A two-stage attention framework has been proposed for facemask-aware robust facial expression recognition. In the first stage, both masked and unmasked images are used to train a binary-classified convolutional network, which generates masked attention heatmaps [23, 25, 26]. In [27], 99.63% of accuracy had been achieved in training masked and unmasked faces, and 90.35% of testing accuracy. In the second stage, the AWFM method is used to generate facemask awareness, then extract facial features, and finally, the FER DEEP classifier to find the final emotion [27]. To train the proposed FER deep classifier, the paper used the M-LFW-FER and M-KDDI-FER datasets, the ratio of the training set to the validation set was 7:3 for each dataset [27]. The result of the performance evaluation of the FER deep classifier is shown in Table 2 [27].

**Table 2:** Performance evaluation of the FER deep

Target	M-LFW-FER		K-DDI-FER	
	Validation	Test	Validation	Test
VGG19	60.17%	47.12%	68.71%	46.21%
Mobile Net	72.18%	52.08%	73.94%	48.71%
RAN	84.72%	79.20%	89.41%	87.45%
ACNN	86.69%	82.53%	88.10%	86.14%
OADN	88.63%	84.21%	91.49%	88.92%

Bo Yang et al. evaluated the performance of two deep learning models, MobileNet and VGG19, for facial expression recognition with three categories (positive, negative, and neutral) and five facial orientations (up, left, center, right, and down) on the masked FER dataset [18]. The results showed a decrease in test accuracy.

This paper presents a model for recognizing facial expressions from both occluded and non-occluded faces. The first approach extracts features from non-occluded faces using the Dlib library and recognizes the emotions of these faces using CNN and LBP. The second approach has observed that in the case of occlusion of part of the face, missing discriminative information due to occlusion only accounts for a very small part of the performance drop. This approach extracts features and recognizes emotion from the remaining exposed part of the face which are described in the next section.

## Methodology

**Fig. 1:**
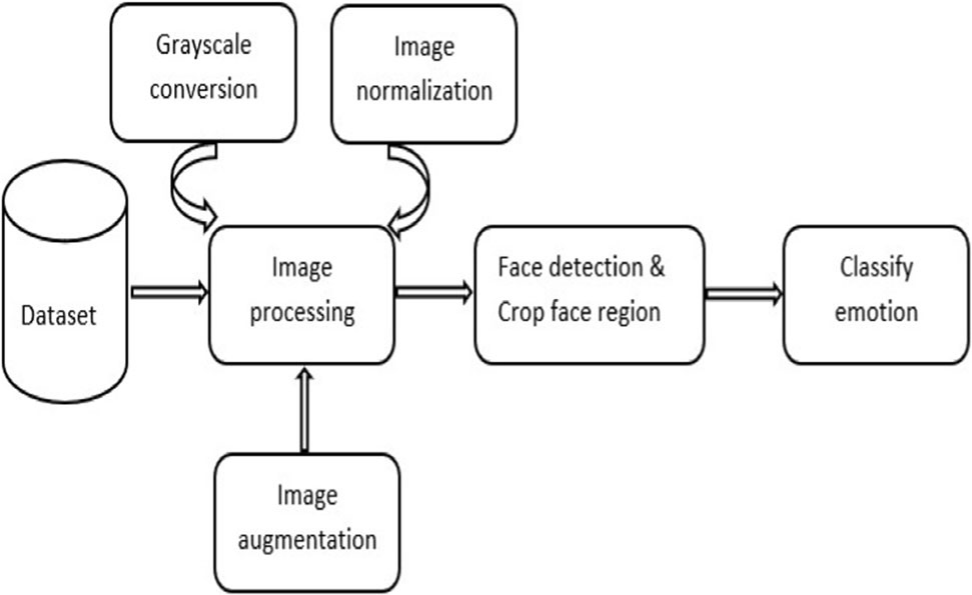
Overall system block diagram

This section introduces the data used for training and testing, explains how the data are preprocessed, and presents the proposed model for facial expression recognition with and without a facemask. Identifying emotions accurately when a person is wearing a mask is challenging, and the accuracy is relatively low. Emotion identification accuracy is relatively low when using a mask. In this paper, CNN is used with data augmentation. The dataset was collected from various sources, which resulted in some variations. However, our proposed model is not affected by these variations [10]. Figure 1 illustrates the steps of our model.

### Dataset preprocessing

The CK + dataset has 327 images with 7 emotions divided as follows: 45 anger, 18 contempt, 59 disgust, 25 fear, 69 happiness, 28 sadness, and 83 surprises [18, 28]. Furthermore, the M-LFW-FER dataset contains 4757 images with 3 different emotions: 2538 positive, 423 negatives, and 1796 neutral [23, 27]. This dataset considers front- and side-view orientations. The front view only contains (positive (2078), negative (338), and neutral (1268)) [27]. The ratio of the training set to the validation set is 7:3 for each dataset. Data augmentation is a method of artificially creating new training data from existing data [10]. To create new and diverse training examples, we use domain-specific techniques to transform examples from the training data. After applying these approaches, the CK + dataset contains 4250 images belonging to seven classes in the training dataset. Before training the model, we preprocess each image by applying face detection, face registration for handling pose variations, and illumination correction based on the locations of extracted facial landmarks. The ImageDataGenerator function of the Keras API was used to supplement the data. Five operations have been included as parameters for the ImageDataGenerator function: rotation at a specific angle, shearing, zooming, horizontal flip, and rescale [10, 29, 30].

Table 3 lists the proposed parameters along with their values. To make all the images of the same size, an image resizing operation is conducted [30].

**Table 3:** Data augmentation parameters

Operation type	Value
Rotation	10
Rescale	1./255
Shear range	0.15
Zoom range	0.1
Horizontal flip	True

The input image is a preprocessed face with dimensions of 48 × 48 pixels and three channels for red, green, and blue. To reduce the complexity of pixel values, dataset images have been converted into grayscale, resulting in only one channel [17]. Normalization has been applied to the model dataset which is a process that modifies the range of pixel intensity values to a certain limit. At last, the augmented dataset is fed into CNN to predict the class [10, 30]

### Face detection and extraction

Face detection could be achieved with many algorithms that detect face location from an image. It employs machine learning algorithms that find, capture, store, and analyze face regions in order to match them with images of individuals in a pre-existing database. Face detection is introduced from the OpenCV method. Face detection is one approach to image processing [31]. The face detection methods we will be covering available in python are OpenCV Haar Cascade, Dlib HoG, OpenCV Deep Learning-based Face Detection, Dlib Deep Learning-based Face Detection, and Mediapipe Deep Learning-based Face Detection. This paper used Dlib HoG Face Detection based on HoG (histogram of oriented gradients) and SVM (support vector machine) and is significantly more accurate than the previous one. It has fast processing algorithm to determine 68 points of landmarks on faces [18].

Dlib is a facial landmark detector with pre-trained models. It is used to estimate the location of 68 coordinates (*x*, *y*) that map the facial points on a person's face [18, 32]. Dlib is used to detect faces due to its fast processing speed. However, the speed of Dlib face detection depends on the size of the image being processed. For larger images, it may take more than 60 milliseconds, but in general, face detection can be done in 15 to 60 milliseconds [32].

**Fig. 2:**
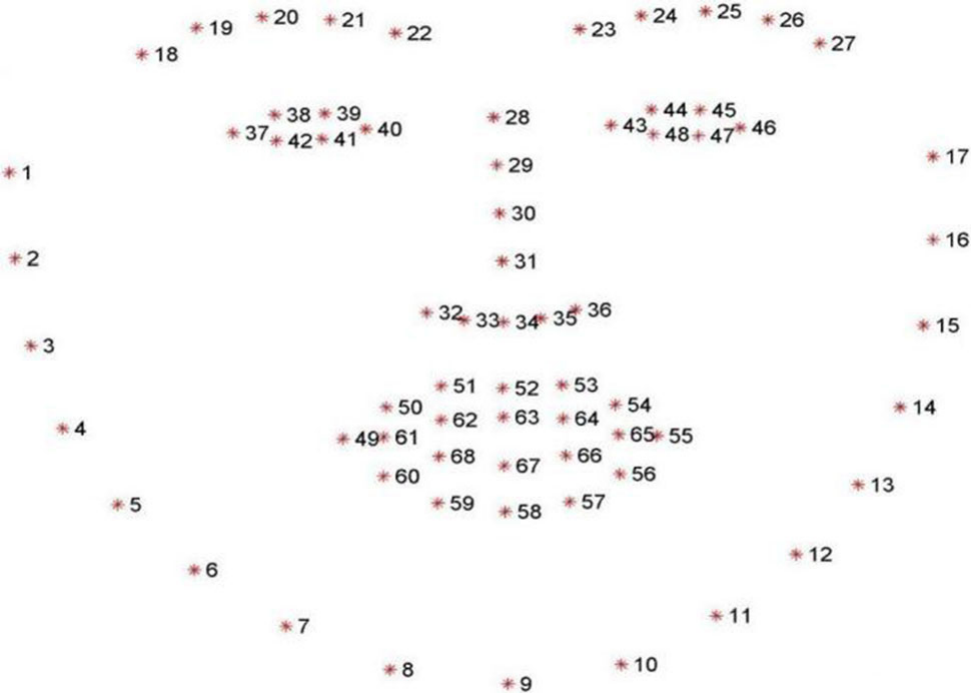
Facial landmarks points with Dlib library [32, 34]

The Dlib library contains facial landmarks with the pre-trained model. This model marks 68 points on the face, and the points specify regions of the face. The jaw line is highlighted using points 1–17, the left eyebrow is highlighted using points 18–22, the right eyebrow is highlighted using points 23–27, the left eye region is highlighted using points 37–42, the right eye region is highlighted using points 43–48, the region of the nose is highlighted using points 28–36, the outer lip area is highlighted using points 49–60, and the inner lip structure is highlighted using points 61–68 [18, 32]. Figure 2 shows landmark points extracted by the Dlib library [32]. This face detection algorithm is appropriate for obtaining exact facial regions, and points 1–27 are used to extract the face region from the original images.

### Local binary pattern

**Fig. 3:**
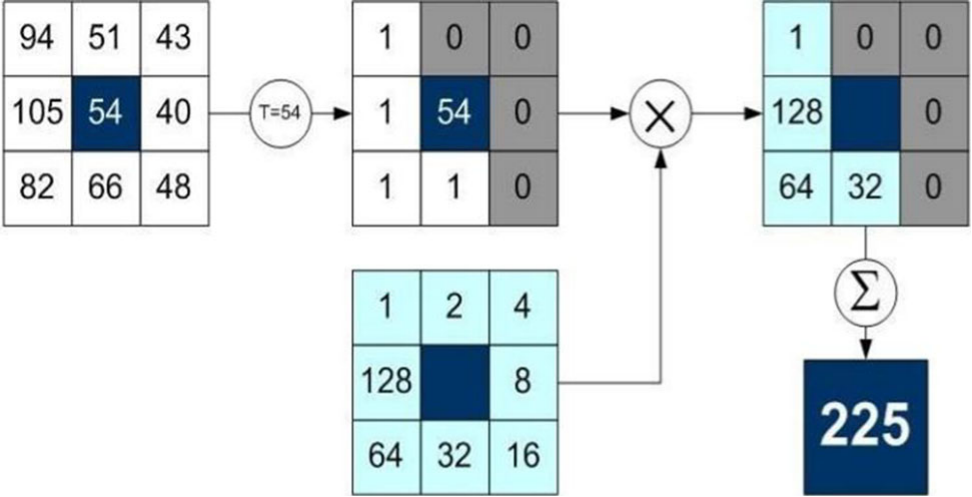
An example of the basic LBP operator [35]

The LBP (local binary pattern) approach has been employed in several applications, including the recognition of human faces and facial expressions using the LBP algorithm [33]. The LBP histograms are derived from a human face Gabor map. After that, the histograms are combined into a single vector. The vector is regarded as a pattern vector [18]. The LBP feature descriptor is widely employed as a reliable illumination invariant feature descriptor. The operator generates a binary number by comparing neighboring pixel values to the center pixel value [15]. The LBP operator is defined for 3× 3 neighborhoods, where each pixel is taken as the central pixel, and the 8 pixels around it are evaluated based on a given threshold [18, 33]. The 8 pixels around each pixel are generated by the bits associated with the local adjacent matrix. These 8 bits are combined to create a binary number, which is subsequently translated to decimal [18]. Fig 3 shows the LBP descriptor. To calculate the LBP code, for each pixel (*p*), the 8 neighbors of the center pixel are compared with the pixel (*p*), and the neighbors *x* are assigned a value of 1 if *x* is greater than (*p*) [35]. A binary number is obtained by concatenating all these binary codes in a clockwise direction starting from the top-left one. To deal with different scales, the LBP operator was later propagated to use neighborhoods of different sizes [35].

A local neighborhood is sampling points evenly spaced on a circle that is centered at the pixel to be labeled, and the sampling points do not drop the pixels are entered, so it allows for any radius of sampling points in the neighborhood. The LBP value is computed by applying scalar multiplication between the binary and weight matrices. Finally, the sum of all multiplication results is used to represent the LBP value. Therefore, LBP value of the matrix 3 × 3 is shown in Fig 3. For center pixels that are on the borders, its neighboring pixels outside of the image can be found by wrapping around the image in a clockwise direction so LBP can be computed as follows: 2^0^ + 2^5^ + 2^6^ + 2^7^ = 1 + 32 + 64 + 128 or equal to 225 [36].

The basic LBP operator is grayscale transformations intensity order in the local neighborhoods [15]. An example of the basic LBP operator is shown in Fig 3 [35]. LBP has been developed for improved performance in different applications, and it does the following features: (1) improve its capability; (2) enhance its robustness; and (3) select its neighbor. An LBP is said to be uniform if the circular binary pattern (clockwise) is made up of at most two bitwise transitions from 0 to 1 when the bit pattern is considered circular [36]. For example, the patterns 00000000 (0 transitions), 00001111 (1 transition), 01110000 (2 transitions), and 11001111 (2 transitions) are uniform, whereas the patterns 11001110 (3 transitions) and 11001001 (4 transitions) are not [37]. For the expression recognition challenge, LBP maps can be utilized to identify the most discriminative features [36].

### The proposed hybrid LBP-CNN model

This study considers several models to select the best approach for facial expression recognition, including feed forward neural network, simple convolution neural network, convolution neural network, and hybrid LBP-CNN. To improve CNN training, this paper proposes a method that incorporates local texture features extracted by LBP, which are more informative than pixel-level features. This paper presents a method of facial expression recognition with and without a mask based on LBP and CNN. The CNN is trained by the LBP feature map of the image as input of CNN. The architecture of CNN is different when working on the M-LFW-FER dataset and CK + dataset.

**Fig. 4:**
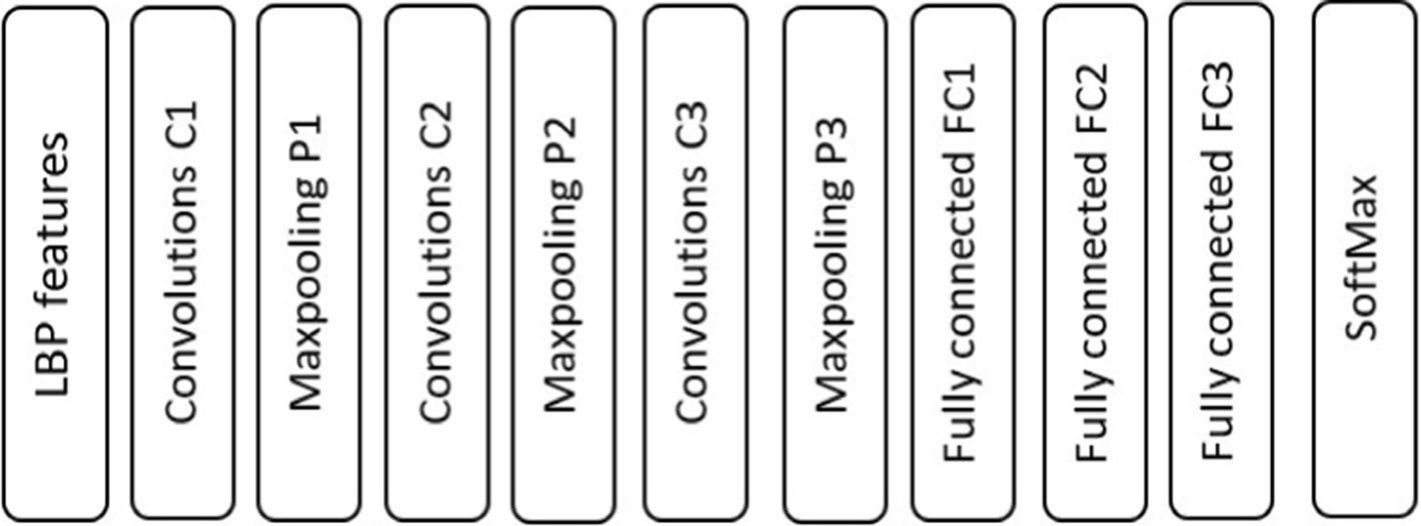
Hybrid LBP-CNN architecture on CK + dataset

**Fig. 5:**
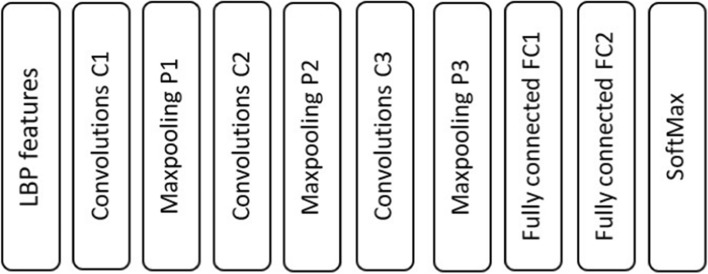
Hybrid LBP-CNN architecture on M-LFW-FER dataset

Figure 4 shows the block diagram of hybrid LBP-CNN architecture on the CK + dataset. Figure 5 shows the block diagram of hybrid LBP-CNN architecture on the M-LFW-FER dataset. The ultimate result of facial expression recognition with and without a mask will be discussed in the next section.

## Experimental results and discussions

This paper uses Colab Pro for training and testing. Colab Pro or “Colaboratory” allows the use of python in the browser with (1) a high guarantee of resources.

(2) K80, T4, and P100 GPUs.

(3) 32 GB RAM.

(4) 24-h runtime.

Keras is used to build neural networks, with a TensorFlow backend running in Python.

Experiments on the CK + and M-LFW-FER datasets are used to evaluate the proposed models. After gathering the data, the faces are recognized, processed, and inputted into the model. Experiments are conducted assuming four different models: feed forward neural network, simple convolution neural network, convolution neural network, and hybrid LBP-CNN. The experiments are run twice: on the CK + dataset without a mask and on the M-LFW-FER dataset with a mask. The CK + dataset is split into 85% training and 15% tests. The model achieved 86.65% test accuracy when a convolution neural network is used only. The M-LFW-FER dataset is split into 80% training, 10% validation and 10% tests. The test dataset used for model evaluation is manually collected images of masked faces from the internet by searching keywords “happy, face, mask” and “angry, face, mask”, and the test dataset was used for testing as a fair evaluation [23].

**Fig. 6:**
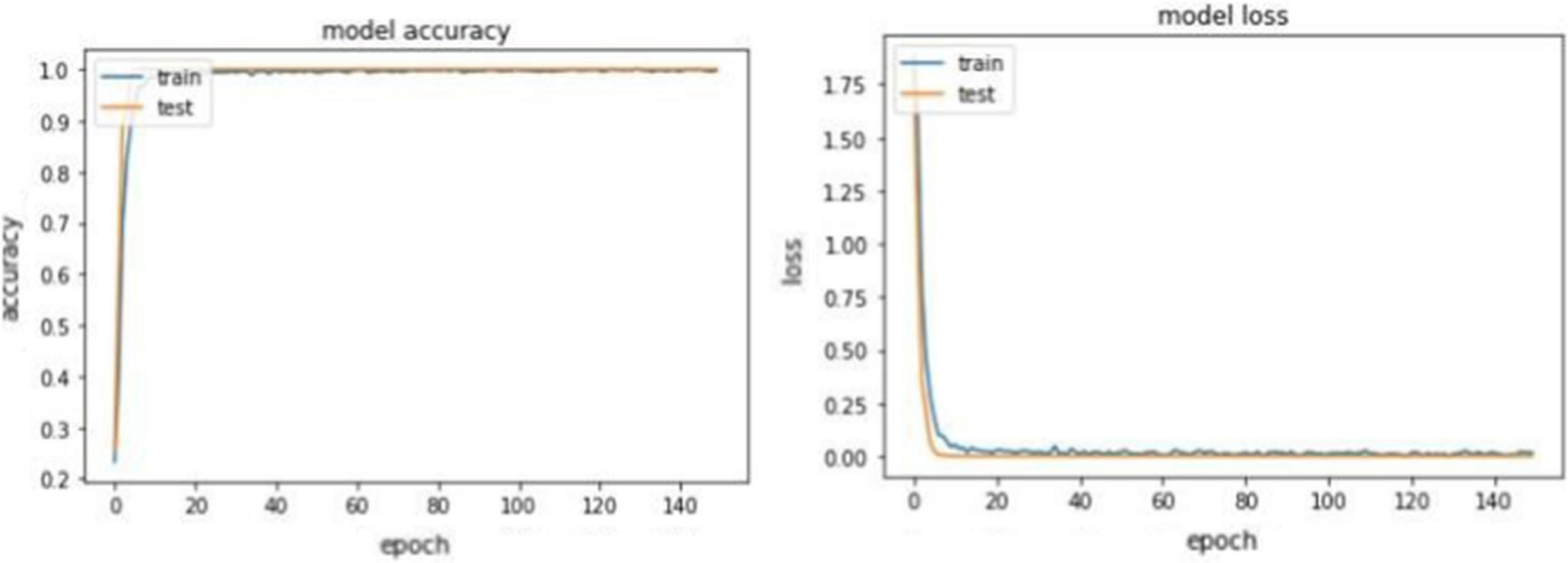
Accuracy and loss curves of hybrid LBP-CNN model on CK + dataset

By extracting features from the input data, feature extraction improves the accuracy of learned models. The test accuracy improved when applying local binary pattern feature extraction and then choosing the best model of CNN (hybrid LBP-CNN model). A comparison of all the models can be shown in Table 4. The bold result in the table is the highest accuracy. Thus, it achieved 90.20% accuracy, which is the highest result for test accuracy. Figure 6 illustrates the learning and loss curves, respectively, for the hybrid LBP-CNN model. The M-LFW-FER dataset with face mask contains three emotions (positive, negative, and neutral). The ratio of the training set to the validation set is 7:3 for each dataset. This dataset contains faces with different orientations: the front- and side-view masks. Results are shown in Table 5. The bold result is the highest result achieved of the proposed models. Compared with other methods, LBP is more effective to extract facial expressions. The LBP and CNN are combined, and the experimental results of hybrid LBP-CNN which is based on CK + and M-LFW-FER datasets showed that our method can enhance the accuracy of AFER, and it can improve the discriminative ability. The result of the dataset which contains front- and side-view faces is worse compared with the dataset which contains front view only. The results were achieved by applying the three proposed models: CNN (front + side view), CNN (front view only), hybrid LBP + CNN (front + side view), and hybrid LBP + CNN (front view only) models on the M-LFW-FER dataset which were 66.37%, 67.84%, 66.80%, and 70.76%, respectively.

**Table 4:** A comparison of all models on CK + dataset

Model	Test accuracy
Feed-forward neural network [38]	78.36%
Simple CNN [38]	80.303%
CNN [17]	86.61%
LBP + CNN [17]	90%
**Proposed CNN**	**86.65%**
**Hybrid LBP** + **CNN**	**90.20%**

The font type has been changed to bold due to the fact that the two results represent the highest accuracy when using the CK+ dataset

**Table 5:** Result of facial expression recognition with a face mask on the M-LFW-FER dataset

Models	Test accuracy
VGG19 [18, 17]	47.12%
Mobile Net [18, 17]	52.08%
RAN [18]	79.20%
ACNN [18]	82.53%
OADN [18]	84.21%
**Proposed CNN** (front + side view)	**66.37%**
**Proposed hybrid LBP** + **CNN** (front + side view)	**66.80%**
**Proposed CNN** (front view only)	**67.84%**
**Proposed hybrid LBP** + **CNN** (front view only)	**70.76%**

Bold font was used as these results represent the highest accuracy when using the M-LFW-FER dataset

The result of CNN_LBP on (front and side view) shows the test accuracy is 66.80%. The decrease in accuracy when applying CNN_LBP on both front- and side-view datasets compared to only the front-view dataset could be due to several reasons. One reason could be that the LBP feature extraction is more effective in capturing the local texture information in front-view images compared to side-view images. Additionally, the CNN model may not be as effective at learning discriminative features from the side-view images as it is from the front-view images, leading to a decrease in overall accuracy. Finally, it is possible that the combined dataset has more class imbalance or noise that could make it more difficult for the model to learn accurate representations.

**Fig. 7:**
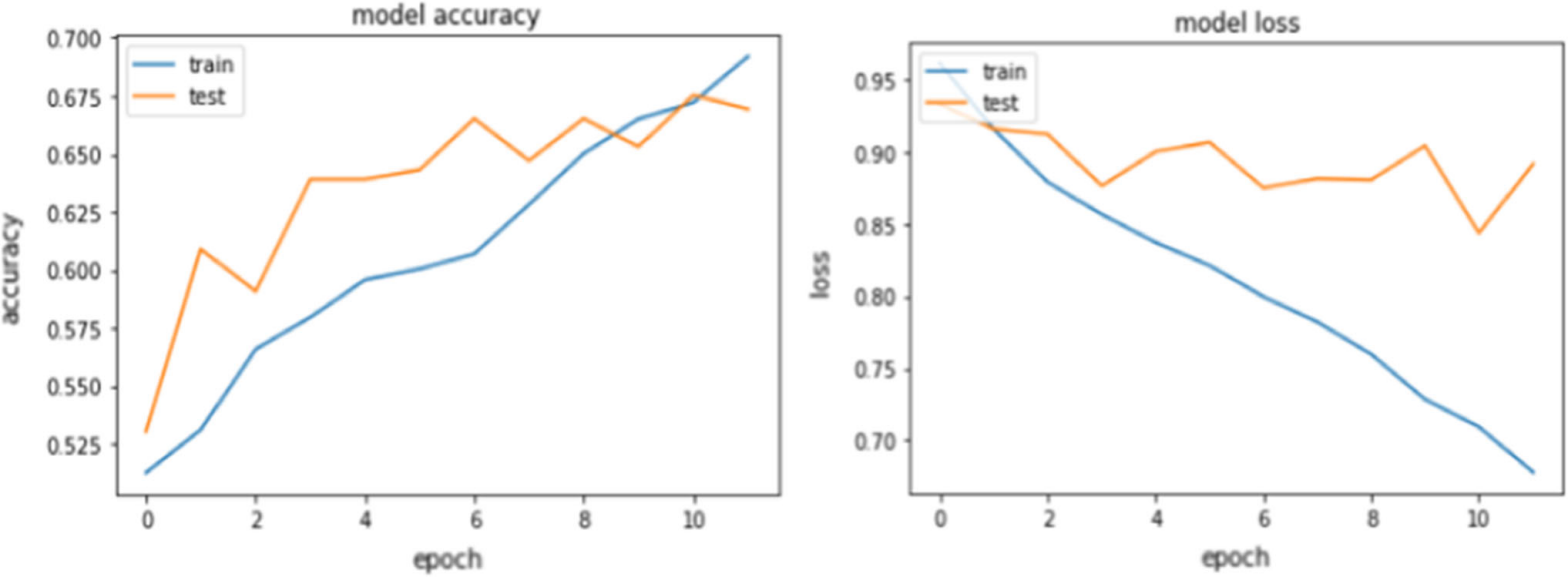
Accuracy and loss curves of proposed CNN (front + side view) model on M-LFW-FER dataset

**Fig. 8:**
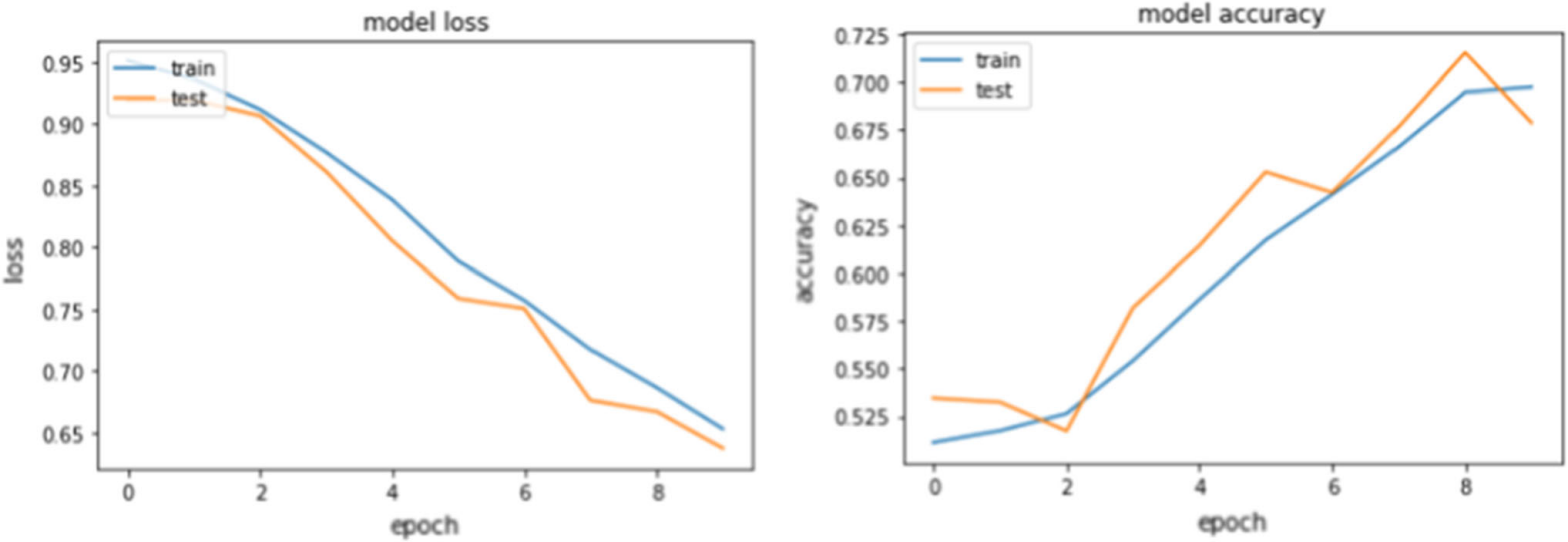
Accuracy and loss curves of proposed CNN and LBP (front + side view) model on M-LFW-FER dataset

**Fig. 9:**
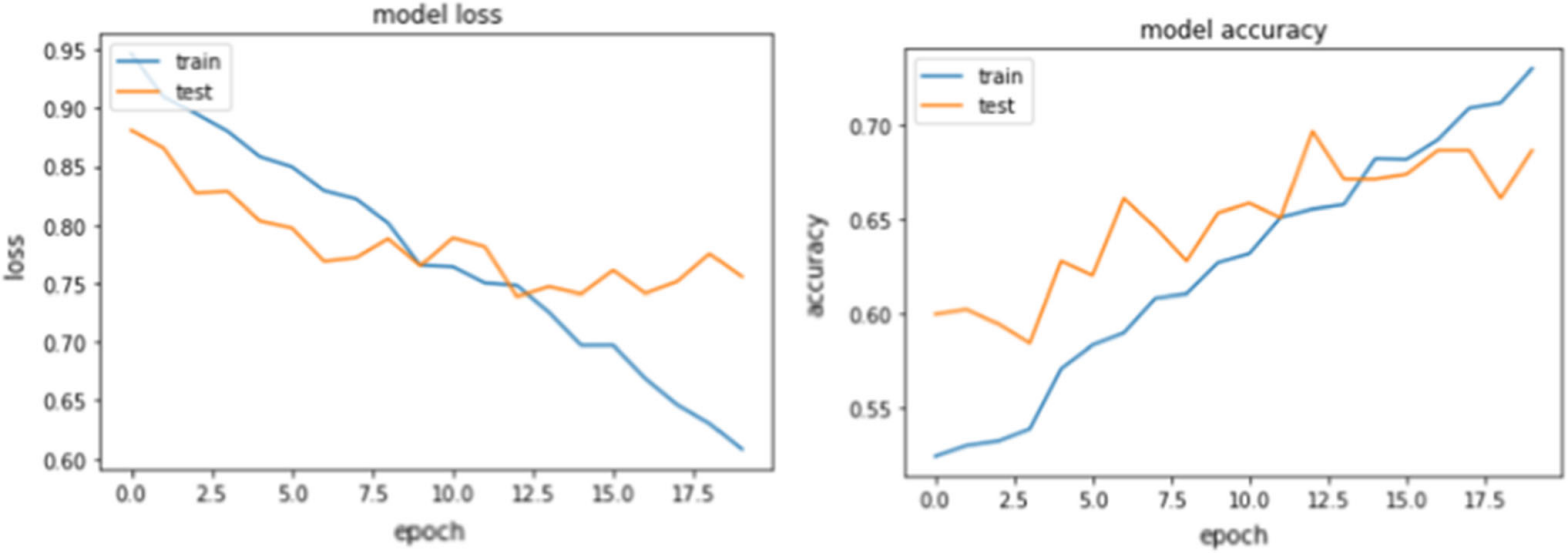
Accuracy and loss curves of proposed CNN (front view only) model on M-LFW-FER dataset

**Fig. 10:**
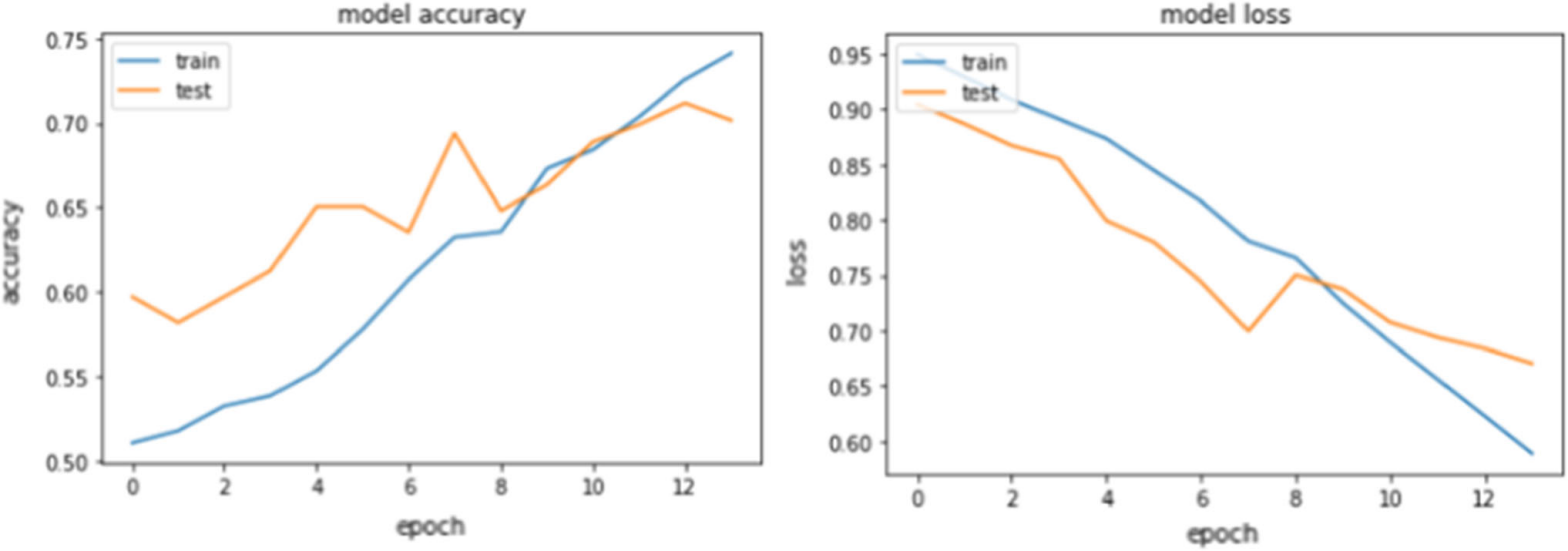
Accuracy and loss curves of proposed CNN and LBP (front view only) model on M-LFW-FER dataset

The training, validation, and testing results for the three proposed models are presented in Table 6. The model accuracy and model loss of the four proposed models are shown in Figs. 7, 8, 9, and 10. This paper introduced different models on two datasets: CK + and M-LFW-FER datasets. The first stage involved the classification of CK + dataset without applying LBP and the test set, which produced an overall accuracy rate of 86.65%. In the second stage, classification after applying LBP gave an overall accuracy rate of 90.20%. On the other hand, the M-LFW-FER dataset is proposed in the first stage classification without applying LBP. This dataset contains front- and side-view images, and the test accuracy is achieved at 66.73%. After applying LBP on M-LFW-FER dataset, the accuracy is 66.80%. In the second stage, when removing side-view images of the M-LFW-FER dataset, the test accuracy produced an accuracy rate of 67.84%. In the third stage of the experiment, classification after applying LBP and the results show an overall recognition rate of 70.76%.

**Table 6:** The train, test and validate accuracies on the three proposed models on M-LFW-FER dataset

Models	Train	Validation	Test
Proposed CNN (front + side view)	75.12%	71.57%	66.37%
**Proposed hybrid LBP** + **CNN** (front + side view)	69.76%	67.89%	66.80%
Proposed CNN (front view only)	72.97%	68.62%	67.84%
Proposed hybrid LBP + CNN (front view only)	74.13%	71.17%	70.76%

The bold font is a response to a question that was asked during the major revision stage

The accuracy of the test dataset is decreasing when facial expression recognition with the mask is applied to the CK + dataset that wearing a face mask. Hence, it became so difficult to achieve better performance on the CK + dataset with a mask. It achieved 36.36% accuracy on seven emotions. This paper compares the performance of AFER systems (Face + +) with human emotion recognition on the M-LFW-FER dataset [39]. Face + + is allowed to analyze faces in images with functions such as face detection and face emotion. Face + + provides probability scores for neutral, anger, sadness, disgust, fear, happiness, and surprise. It achieved 35.63% accuracy on three emotions (neutral, positive, and negative) of the M-LFW- FER dataset (masked faces).

## Conclusion

In this research, CK + and M-LFW- FER datasets were preprocessed. The CK + dataset comprises seven face emotions without a face mask, while the M-LFW-FER dataset comprises three emotions with a face mask. This paper introduced two models: the first was a CNN, while the second was a hybrid LBP-CNN. Obtained results showed that the M-LFW-FER (masked faces) achieved a relatively lower detection accuracy compared to CK + (unmasked faces); which means that the masked face has decreased the performance. The proposed CNN reached an accuracy of 86.65%. Four experiments were run for masked faces on the M-LFW-FER dataset: CNN with front-view, CNN with front- and side-views, LBP-CNN with front and side view, and LBP-CNN with front view. Test accuracies changed to 67.84%, 66.37%, 66.80%, and 70.76%, respectively.

Future research could focus on developing technologies or algorithms that can accurately detect facial expressions and emotions based on upper part of the head when a person is wearing a mask. This could involve using computer vision techniques to analyze subtle changes in the eyes or other visible parts of the face and also incorporating hand or body movements to identify emotions.

## Data Availability

The datasets used during the current study are available from the corresponding author upon reasonable request.
